# The Absence of Typical Stroke Symptoms and Risk Factors Represents the Greatest Risk of an Incorrect Diagnosis in Stroke Patients

**DOI:** 10.3390/jpm14090964

**Published:** 2024-09-11

**Authors:** Rakesh Jalali, Aleksandra Bieniecka, Marek Jankowski, Patryk Stanisław Michel, Marta Popielarczyk, Mariusz Krzysztof Majewski, Jacek Zwiernik, Joanna Maria Harazny

**Affiliations:** 1 Department of Emergency Medicine, School of Medicine, University of Warmia and Mazury, 10-719 Olsztyn, Poland; 2 Students’ Research Group, Department of Human Physiology and Pathophysiology, School of Medicine, University of Warmia and Mazury, 10-719 Olsztyn, Poland; aleksandra.bieniecka@student.uwm.edu.pl (A.B.); 158580@student.uwm.edu.pl (M.J.); patryk.michel@student.uwm.edu.pl (P.S.M.); 3 School of Public Health, University of Warmia and Mazury, 10-719 Olsztyn, Poland; 146986@student.uwm.edu.pl; 4 Department of Human Physiology and Pathophysiology, School of Medicine, University of Warmia and Mazury, 10-719 Olsztyn, Poland; mariusz.majewski@uwm.edu.pl (M.K.M.); joanna.harazna@uwm.edu.pl (J.M.H.); 5 Department of Neurology and Neurosurgery, School of Medicine, University of Warmia and Mazury, 10-719 Olsztyn, Poland; jacek.zwiernik@uwm.edu.pl; 6 Clinical Research Center, Department of Nephrology and Hypertension, University Erlangen-Nuremberg, 91054 Erlangen, Germany

**Keywords:** stroke, stroke risk factors, emergency medicine, neurological stroke symptoms, incorrect diagnosis

## Abstract

Background: Stroke is one of the most misdiagnosed conditions that causes serious medical disabilities. Its early and accurate diagnosis by the emergency team is crucial for the patient’s survival. This study aimed to determine the percentage of brain strokes incorrectly diagnosed by paramedic teams and to analyze the factors influencing incorrect diagnoses. Methods: The data of 103 patients, mean age of 68.4 ± 14.96 years, admitted in 2019 to hospital emergency departments of the two hospitals in Olsztyn, Poland, were analyzed retrospectively. All patient data were obtained from their information cards. The parameters of the patients misdiagnosed and accurately diagnosed by paramedics were analyzed with Odds Ratio (OR) calculations using IBM SPSS version 23 software. Results: Stroke and transient ischemic attack were recognized in 77 cases (74.8%). In 26 patients (25.2%), the diagnosis made in the ambulance differed from that made in the hospital ward. The analysis of the Odds Ratio (OR) has shown that typical stroke risk factors, if present in a patient, facilitate the correct diagnosis. The greatest source of misdiagnosis of stroke by the paramedic team was the lack of hemiplegia (OR = 6.0). Conclusions: The absence of typical stroke risk factors and neurological stroke symptoms, such as smoking, hemiplegia, aphasia, hypercholesterolemia, arrhythmia, diabetes or a drooping corner of the mouth, constitutes a high risk of misdiagnosing stroke by the paramedic team.

## 1. Introduction

Stroke remains the second leading cause of death and, when adjusted for disability-adjusted life years (DALYs), the third leading cause of death and disability combined worldwide [1,2]. Shortly before the SARS-CoV-2 pandemic in 2019, 81,182 patients were admitted to hospitals in Poland due to stroke. Data from 2010 to 2019 reveal that 86.2% of those hospitalized for stroke had an ischemic stroke (IS) and 13.8% had a hemorrhagic stroke (HS) [3].

Paramedics have to rely on the patient’s medical history, risk factors and physical examination to diagnose a stroke. Despite having a variety of different stroke diagnosis tools, such as the Face, Arms, Speech, Time (FAST) test or Cincinnati Prehospital Stroke Scale, in a considerable number of cases the primary diagnosis does not match the actual condition of the patient [4]. According to many studies, stroke in a large number of patients is suspected based on stroke mimics [5,6,7,8,9,10,11].

It is up to the paramedic team to determine the exact time that has elapsed since the onset of symptoms. This is important because it affects the eligibility of patients for thrombolytic therapy, and a prompt administration of recombinant tissue plasminogen activator (rt-PA) increases its benefit. Early recognition is associated with markedly shorter time until arrival at the stroke unit and it is a key to a successful treatment [4,12].

The aim of this study was to determine the percentage of incorrectly diagnosed strokes by paramedics and to analyze the risk factors contributing to a misdiagnosis.

## 2. Materials and Methods

### 2.1. Design and Study Population

The study was a retrospective cohort analysis. The retrospective study “The lack of stroke risk factors was the risk of incorrect diagnosis of stroke patients by the paramedics in 2019 in Olsztyn”, based on decision no. 2/2024 of the Ethics Committee of the University of Warmia and Mazury in Olsztyn, which operates in accordance with the principles of Good Clinical Practice and applicable law, did not require the consent of the Bioethics Committee for this type of research. The data of patients admitted to emergency departments (EDs) in two hospitals in Olsztyn, Poland, in 2019, in whom paramedics suspected a stroke, were analyzed. This was the last year in Olsztyn before the SARS-CoV-2 pandemic, which excluded the impact of this virus on the study results. The study population included 103 patients: 49 from the Ministry of Internal Affairs and Administration Hospital (the MSWiA Hospital) in Olsztyn and 54 from the Regional Specialist Hospital (the WSS Hospital) in Olsztyn, aged 23 to 97, with a mean age of 68.4 ± 14.96 years. There were 49 women (47.6%) and 54 men (52.4%). The managing directors of both hospitals provided written consent to use patient files to conduct the analyses. The data were encoded by generating individual patient ID codes before analysis.

### 2.2. Imaging in Stroke Patients

Both hospitals participating in the study had a CT scanner as well as an MRI scanner. The decision to perform a specific examination was made by a neurologist. As is standard, a CT scan without contrast was performed, and in the case of a high NIHSS score, it was supplemented with an angioCT scan of the cerebral arteries, and in the case of possible mechanical thrombectomy, a post-processing assessment of cerebral perfusion was performed using the Brainomix system. MRI was performed in patients with wake-up stroke symptoms and in doubtful cases suggesting, for example, posterior circulation stroke. If the CT or MRI scan performed on the day of admission did not show a stroke, then after 48–72 h, an MRI scan was performed if possible or CT if there were contraindications to MRI.

### 2.3. Data Collection

The data were retrieved from the medical archives of both hospitals. The criteria for including patients in the analyses were a diagnosis of “stroke” without a stroke specification made by paramedics and patients with a final diagnosis (ischemic or hemorrhagic) or transient ischemic attack (TIA) made in the hospital ward. No patients were excluded from either group. All patient data were obtained from patients’ information cards, medical rescue cards, hospital ED cards and hospital discharge cards. The collected data included: gender; place of residence (urban or rural area); age; clinical signs (consciousness, visual disturbances, anisocoria, hemiplegia, aphasia, a drooping corner of the mouth or orientation) and the side of their occurrence, if applicable; initial prehospital and hospital discharge diagnoses (ICD-10); diagnosis consistency (correct or incorrect); presence of comorbidities; time since the last stroke; vital parameters measured before admission to the hospital ward (SBP—systolic blood pressure, DBP—diastolic blood pressure, MAP—mean arterial pressure, PP—pulse pressure, HR—heart rate, SaO2—oxygen saturation, RR—respiratory rate, BGL—blood glucose level); smoking; hospitalization ward; and death (yes or no). No sensitive data were included in the tables. 

### 2.4. Data Analysis

Statistical analyses were performed with the use of the IBM SPSS Statistics version 23 software (IBM, New York, NY, USA). The Kolmogorov–Smirnov test was used to test the normality of variable distributions. To compare the means of the analyzed parameters with the normal distribution (age, PP) between the groups of patients, the *t*-Student test was used. For unpaired measurements with a non-normal distribution of results (other parameters), the differences in median values were compared with the non-parametric U-Mann–Whitney test. Correlation analyses were performed using Pearson’s correlation coefficient and Spearman’s correlation coefficient in the case of non-normal distribution. The Odds Ratio (OR) was calculated for the analyzed risk parameters of an incorrect diagnosis by paramedics. The description of the OR calculation can be found in the Supplementary Materials.

To determine significant differences (p_chiQ_) in the categorical binary variables (yes/no), a Chi-Square test was used.

The result of the analyses was considered statistically significant at *p* < 0.05.

## 3. Results

### 3.1. Gender Distribution

The study population consisted of 103 patients: 54 males (52.4%) and 49 females (47.6%). The differences between male and female patients were not significant. The only clinical parameter that varied significantly (*p* = 0.04) between genders was RR. The mean RR was 15.17/min ± 2.50/min in females, while in males it was 16.65/min ± 2.75/min. Clinical characteristics of the study population collected by paramedics are presented in Table 1.

### 3.2. Comparison of Parameters Depending on the Place of Further Treatment

SBP was the only clinical parameter significantly (*p* = 0.034) different between patients treated in the MSWiA Hospital and the WSS Hospital in Olsztyn. Patients with higher SBP tended to be transported to and treated in the MSWiA Hospital. The mean SBP for patients hospitalized there was 161.2 mmHg ± 30.5 mmHg, while for patients treated in the WSS Hospital in Olsztyn it was 151.4 mmHg ± 26.0 mmHg. Gender and place of residence were not decisive factors in the distribution of patients to these hospitals. Age was the only parameter that differed between urban and rural residents. Patients with suspected stroke who lived in rural areas were tendentiously (*p* = 0.068) on average 5 years older than those who lived in cities (70.96 years ± 15.23 and 65.57 years ± 14.29, respectively).

### 3.3. Analysis of Prehospital Recognition of Stroke

Comparing the initial ICD-10 diagnosis made by the paramedics to the final ICD-10 diagnosis included in hospital discharge documents, the accuracy of the initial diagnosis was analyzed in two different variants:

(1) The number of entirely dissimilar diagnoses was assessed, i.e., it was analyzed whether the initial diagnosis and the final one referred to the same disease. The diagnosis of unspecified stroke (ICD-10 I64) was considered to match when the final diagnosis was also a specified type of stroke (ischemic or hemorrhagic) or transient ischemic attack (TIA) (Table 2).

According to the data collected, 18 patients initially received an entirely different diagnosis than the one provided at hospital discharge. That group constituted 17.5% of cases. In the remaining 85 cases (82.5%), the initial diagnosis was accurate (Table 2).

The chosen hospital to which the patient was admitted did not affect the accuracy of the diagnoses made by paramedics, which differed from the one made in the hospital setting in all of those cases. The number of patients whose diagnoses differed is placed in brackets in each ICD-10 code category. 

The diagnoses with the number of cases (N) made in the hospital setting that varied from the initial diagnosis of stroke by paramedics (ICD-10 I64) were as follows:

- G45.4 Transient global amnesia (N = 3);
- G45 Transient cerebral ischemic attacks and related syndromes (N = 4);
- G40.1 Localization-related (focal/partial) symptomatic epilepsy and epileptic syndromes with simple partial seizures (N = 1);
- R47.8 Other and unspecified speech disturbances (N = 1);
- G40.9 Epilepsy, unspecified (N = 2);
- Z30.9 Contraceptive management, unspecified (N = 1);
- G44.8 Other specified headache syndromes (N = 1);
- Z03.3 Observation for suspected nervous system disorder (N = 1);
- R47.8 Other and unspecified speech disturbances (N = 2);
- I63.5 Cerebral infarction due to unspecified occlusion or stenosis of cerebral arteries (N = 1);
- In one case, when the paramedics diagnosed a patient with fracture of the skull and facial bones (ICD-10 S02), the final hospital diagnosis was G45.9 Transient cerebral ischemic attack, unspecified (N = 1).

(2) It was assessed how many initial diagnoses of stroke without a precise distinction between the stroke type and TIA were confirmed in the hospital ward [1]. Among 103 analyzed patients, stroke was confirmed in the hospital setting in 62 cases (60.2%) and TIA in 15 (14.6%). A total of 26 cases (25.1%) were not diagnosed as cerebrovascular episodes at all (Table 3).

The analysis of the group diagnosed with TIA revealed a statistically significant difference in assigning patients to the hospitals for further treatment (p_chiQ_ = 0.005), since 13 out of 15 patients with TIA (86.6%) were treated in the WSS Hospital in Olsztyn and only 2 out of 15 (13.33%) were admitted to the MSWiA Hospital. Among patients diagnosed with stroke and other diagnoses, there were no statistically significant differences in terms of choosing the hospital for further treatment (diagnosis of stroke: *p* = 0.43; unrecognized stroke: *p* = 0.20).

### 3.4. Factors Affecting the Diagnosis

The most important analysis performed in the study focused on factors that help recognize stroke in prehospital conditions and those that hinder the correct diagnosis. Diagnostic accuracy was not influenced by factors such as the age and gender of the patients, their place of residence and the hospital that the patient was transported to.

**Figure 1:**
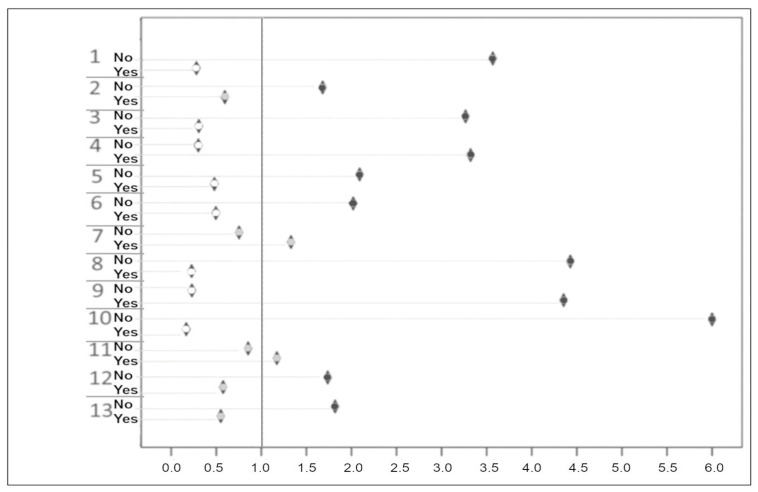
OR of making an incorrect diagnosis by paramedics. 1 smoking; 2 previous stroke; 3 hypercholesterolemia; 4 arrhythmia; 5 arterial hypertension; 6 diabetes mellitus; 7 preserved orientation; 8 drooping mouth corner; 9 aphasia; 10 hemiplegia; 11 visual disturbances; 12 anisocoria; 13 conscious. Circle in diamonds: white—OR ≤ 0.5; gray—0.5 < OR < 1.5; black OR ≥ 1.5.

The study group, i.e., patients who were not diagnosed with stroke by paramedics, consisted of 26 people. The control group, in which the diagnosis of stroke/TIA was made correctly in prehospital care, included 77 patients. The total number of studied cases was 103 for each analyzed risk factor, apart from a drooping mouth corner and aphasia, where the documentation was incomplete in one case. For those factors, the studied population was 102 patients (Table 4, Figure 1).

Based on the analysis of the relative risk of an incorrect diagnosis of stroke or TIA by paramedics, the following observations were recorded:

- The consciousness of a patient was related to an increased risk of an incorrect diagnosis.
- The presence of anisocoria made it two-fold easier to recognize the stroke; however, its absence was related to an increased risk of an incorrect diagnosis.
- Vision deficiencies or their absence did not affect the diagnostic process.
- Hemiplegia occurred in nearly every case in which stroke was correctly recognized and turned out to be the most important factor in arriving at a correct diagnosis of stroke. Absence of hemiplegia was related to the highest risk of a misdiagnosis out of all the considered parameters.
- In the presence of aphasia, paramedics tended to make an incorrect diagnosis of stroke. On the other hand, aphasia was the most important factor that led to an inaccurate diagnosis as in the absence of aphasia the risk of an inaccurate diagnosis was low.
- A drooping corner of the mouth was a clinical sign associated with a high chance of paramedics and hospital staff members making the same diagnosis; the absence of a drooping corner of the mouth significantly increased the risk of an incorrect diagnosis in prehospital care.
- A disturbed orientation of the patient contributed to a misdiagnosis, but a preserved orientation did not influence the diagnostic process.
- If the patient was diabetic and their glucose level was high, a correct diagnosis was made more often.
- Among patients with hypertension, stroke was diagnosed correctly, while in patients with no hypertension, the risk of a misdiagnosis was high.
- Arrhythmia is an excellent indicator of an accurate diagnosis. Its presence facilitated making a correct diagnosis. In most cases, the detected arrhythmia was atrial fibrillation, which is an important risk factor for an ischemic stroke. Absence of arrythmia raised the risk of misdiagnosis.
- Hypercholesterolemia increased the possibility of a correct diagnosis, while the normal level of blood cholesterol in patients was related to a high risk of a mistake in the prehospital recognition of stroke.
- A previous cerebrovascular incident had no influence on the diagnostic process; however, if it was the first cerebrovascular incident in the patient’s history, the risk of an inaccurate diagnosis was high.
- If a patient was a cigarette smoker, the chances of a correct diagnosis were high, while being a non-smoker lowered the probability of a recognition of stroke in prehospital care.

## 4. Discussion

Time plays a crucial role in stroke recognition. An inaccurate diagnosis prolongs the time that elapses from the onset of symptoms to hospital admission, and this can disqualify a patient from receiving lifesaving rt-PA that can be administered only within the first 4–5 h and maximally up to 6 h [4,12,13,14].

Our study established that, in 2019, the percentage of correct initial diagnoses made by paramedics in Olsztyn was high and amounted to 82.5%, and in the case of stroke or TIA it was 74.8%. A similar study performed in Poland by Karliński et al. revealed that the work of paramedics in prehospital conditions showed a high diagnostic sensitivity and positive predictive value (PPV), but lower than that of ambulance physicians and higher than that of non-ambulance physicians. A diagnosis made by paramedics was characterized by a sensitivity of 85% and a PPV of 72% [15]. Similar results were obtained in previous studies [16,17,18,19,20,21,22], but it is important to consider the differences in organizational structures of prehospital care among various countries.

The data presented above indicate that the absence of hemiplegia and the presence of aphasia are associated with the highest probability of a misdiagnosis. For example, aphasia was diagnosed to be associated with disorders of consciousness or dementia. This is also reflected in the calculated CI values. Diagnostic accuracy was not influenced by factors such as age, gender, place of residence and the hospital that the patient was transported to. The presence of common risk factors for stroke—such as hypertension, diabetes, arrhythmia, smoking and high cholesterol levels—facilitated an accurate diagnosis. Some of the conditions that paramedics considered a stroke included epilepsy, dementia, aphasia related to mental disorders and other vascular abnormalities affecting large arteries.

The analyses presented in the study raise the questions that need to be addressed for correct diagnosis of stroke, including: how should paramedics correctly recognize a stroke in patients who do not present with typical stroke symptoms? This is an important question because these patients may have a greater chance of surviving a stroke or even making a full recovery if the correct diagnosis is made promptly. In our opinion, prehospital assessment scales, such as FAST, the Los Angeles Motor Scale or the Cincinnati Prehospital Stroke Scale, designed to quickly identify patients eligible for thrombolytic therapy, rely mainly on symptoms resulting from circulatory disorders in the middle cerebral artery (MCA) or internal carotid artery (ICA) vascularization. These scales neglect the symptoms of posterior circulation stroke and do not help in detecting symptoms of lacunar stroke. On the other hand, paramedics who deal with patients with many different conditions daily may have difficulty mastering more complex assessment scales, such as the National Institutes of Health Stroke Scale (NIHSS) [23,24].

Additionally, a high workload causes burnouts and a tendency for paramedics to think schematically, making it easier for them to suspect stroke in patients with typical stroke symptoms. In our opinion, in addition to searching for more sensitive clinical assessment scales, the continuous education of paramedics is important [25,26]. In our centers, this is achieved by performing a neurological examination of the patient, carried out by a neurologist already present in the ED at the time of patient’s arrival, in the presence of paramedics and providing information to paramedics about the final diagnosis. While conducting this study, mandatory training for paramedics was organized in Poland by the Ministry of Health under the name of Good Practices in Managing a Patient with Suspected Stroke, which unified the standard of practice for paramedics https://www.gov.pl/web/zdrowie/dobre-praktyki-postepowania-z-pacjentem-z-podejrzeniem-udaru-mozgu (accessed on 31 December 2019).

### Limitations

The limitation of this work is that it is focused on analysis of the risk of incorrect diagnosis when diagnosing patients with suspected stroke by paramedics. In our study, the subgroup of patients with an incorrect diagnosis by paramedics was particularly small for a detailed comparative analysis of neurological symptoms and stroke risk factors in patients. In the future, the scope of these analyses could be deepened in a larger research group. The impact of the SARS-Cov-2 pandemic could also be taken into account in subsequent studies, including multicenter studies. The Supplementary Materials include tables with the results of analyses of neurological symptoms and stroke risk factors in the entire study group of patients and in subgroups with correct and incorrect diagnosis by paramedics. A significant limitation of these studies is the lack of information about stroke patients who were not diagnosed with stroke by paramedics and were not transported to hospital for further evaluation. This limitation should be addressed in future studies.

## 5. Conclusions

The absence of typical risk factors and symptoms for stroke in a patient, such as smoking, hemiplegia, aphasia, hypercholesterolemia, arrhythmia, diabetes or a drooping corner of the mouth, constituted a high risk of stroke misrecognition by paramedics in 2019 in Olsztyn. Development of more sensitive scales for stroke assessment is required for eliminating stroke misdiagnosis.

## Figures and Tables

**Table 1:** Clinical characteristics of the study population collected by paramedics.

Parameter	N	Min	Max	Mean	SD	Median	IQR (25, 75)
Age [years]	103	23	97	68.40	14.96	70.00	(58, 78)
SBP [mmHg]	103	100	260	156.04	28.49	155.00	(137, 174)
DBP [mmHg]	103	54	150	87.72	17.25	88.00	(80, 95)
MAP [mmHg]	103	43	167	110.49	18.88	107.33	(98.33, 120.67)
PP [mmHg]	103	20	160	68.32	22.48	70.00	(50, 80)
HR [1/min]	103	55	150	83.60	17.71	80.00	(70, 94)
SaO_2_ [%]	84	90	99	94.17	13.,87	97.00	(95, 98)
RR [1/min]	72	10	24	15.93	2.72	16.00	(14, 18)
BGL [mg/dL]	73	37	380	140.66	52.80	126.00	(111, 156.5)

SBP—systolic blood pressure, DBP—diastolic blood pressure, MAP—mean arterial pressure, PP—pulse pressure, HR—heart rate, SaO_2_—oxygen saturation, RR—respiratory rate, BGL—blood glucose level, SD—standard deviation, IQR—inter quartile range (25th percentile, 75th percentile).

**Table 2:** Compatibility between the initial (paramedics) and final (hospital) diagnoses.

Correct Diagnosis	n (N = 103)	[%]
yes	85	82.5
no	18	17.5

**Table 3:** Compatibility between the initial and final diagnoses of stroke or TIA.

Diagnosis	n (N = 103)	[%]
Stroke	62	60.2
TIA	15	14.6
CVA mimics	26	25.2

CVA mimics—other diseases that mimic symptoms of a cerebrovascular accident. The clinical parameters did not vary significantly between patients with accurate and inaccurate diagnoses of stroke or TIA.

**Table 4:** Factors influencing the diagnosis process by paramedic members.

	S_yes_	C_yes_	S_no_	C_no_	OR
No hemiplegia	13	11	13	66	6.00
No drooping mouth corner	21	37	5	39	4.43
Aphasia	17	23	9	53	4.35
Non-smoker	11	37	1	12	3.57
No arrhythmia	21	43	5	34	3.32
No hypercholesterolemia	23	54	3	23	3.27
No arterial hypertension	11	20	15	57	2.09
No diabetes mellitus	21	52	5	25	2.02
Conscious	24	66	2	10	1.82
No anisocoria	25	72	1	5	1.74
No previous stroke	21	55	5	22	1.68
Disturbed orientation	15	39	11	38	1.33
Visual disturbances	5	13	21	64	1.17
No visual disturbances	21	64	5	13	0.85
Preserved orientation	11	38	15	39	0.75
Previous stroke	5	22	21	55	0.60
Anisocoria	1	5	25	72	0.58
Unconscious	2	10	24	66	0.55
Diabetes mellitus	5	25	21	52	0.50
Arterial hypertension	15	57	11	20	0.48
Hypercholesterolemia	3	23	23	54	0.31
Arrhythmia	5	34	21	43	0.30
Smoker	1	12	11	37	0.28
No aphasia	9	53	17	23	0.23
Drooping mouth corner	5	39	21	37	0.23
Hemiplegia	13	66	13	11	0.17

S_yes_—study group of patients with stroke symptoms or risk factors but incorrectly diagnosed by paramedics. S_no_—study group of patients with no stroke symptoms or risk factors and incorrectly diagnosed by paramedics. C_yes_—control group of patients with stroke symptoms or risk factors and correctly diagnosed by paramedics. C_no_—control group of patients with no stroke symptoms or risk factors and correctly diagnosed by paramedics. OR—Odds Ratio.

## Data Availability

All the analyzed data have been included in the manuscript.

## Supplementary Materials

The following supporting information can be downloaded at: https://www.mdpi.com/article/10.3390/jpm14090964/s1, Table S1: Significance of chi square differences (pchiQ) in YES/NO responses in the study group of patients. Upper and lower limits of the 95% confidence interval (CI 95%) were calculated using the Monte Carlo method; Table S2: Significance of chi square differences (pchiQ) in YES/NO responses in the subgroups of patients with correct und incorrect diagnosis. Upper and lower limits of the 95% confidence interval (CI 95%) were calculated using the Monte Carlo method.
