# Supplementary material for: The Absence of Typical Stroke Symptoms and Risk Factors Represents the Greatest Risk of an Incorrect Diagnosis in Stroke Patients
Source: J Pers Med. 2024 Sep 11;14(9):964. doi: 10.3390/jpm14090964 (PMC11433094; doi:10.3390/jpm14090964)
Supplement: Supplementary file 1 [file jpm-14-00964-s001.zip › jpm-3124214-supplementary.pdf]

Supplementary Materials:

The Odds Ratio calculation:

The study group (S) was defined as patients with incorrect diagnoses. control group (C) consisted of patients with correct diagnoses made by EMTs. The event occurred if in anamneses the question about a risk parameter was answered with "yes". otherwise as "no". That resulted in the differentiation of the following groups:

Syes – study group of patients with stroke symptoms or risk factors but incorrectly diagnosed by EMTs

Sno – study group of patients with no stroke symptoms or risk factors and incorrectly diagnosed by EMTs

Cyes – control group of patients with stroke symptoms or risk factors and correctly diagnosed by EMTs

Cno – control group of patients with no stroke symptoms or risk factors and correctly diagnosed by EMTs

OR was calculated by the formula: OR = (Syes / Cyes) / (Sno / Cno)

OR carried by each parameter with the value close to 1.0 (from 0.5 to 1.5) indicates a minor influence or no influence at all on the diagnosis. The risk of making an incorrect diagnosis is present for factors with OR above 1.5. A high and significant risk is marked by OR above 2.5. OR value below 0.5 indicates a positive influence on the diagnosis correctness.

6.2. Detailed analysis of the tested parameters

Table S1. Significance of chi square differences (pchiQ) in YES/NO responses in the study group of patients. Upper and lower limits of the 95% confidence interval (CI 95%) were calculated using the Monte Carlo method

Parameters	Answers YES versus NO in all patients			
	p_{chiQ}	CI 95% lower limit	CI 95% upper limit	YES [%]
Smoking	<0.001	0.000	0.000	12.6
Previous stroke	<0.001	0.000	0.000	26.3
Hypercholesterolemia	<0.001	0.000	0.000	25.2
Arrhythmia	0.018	0.015	0.020	37.9
Arterial hypertension	<0.001	0.000	0.000	69.9
Diabetes mellitus	<0.001	0.000	0.000	29.1
Preserved orientation	0.700	0.870	0.705	47.6
Drooping mouth corner	0.196	0.189	0.204	42.7
Aphasia	<0.001	0.000	0.000	68.0
Hemiplegia	<0.001	0.000	0.000	76.7

Visual disturbances	<0.001	0.000	0.000	18.0
Anisocoria	<0.001	0.000	0.000	5.8
Conscious	<0.001	0.000	0.000	87.6

Table S2. Significance of chi square differences (pchiQ) in YES/NO responses in the subgroups of patients with correct und incorrect diagnosis. Upper and lower limits of the 95% confidence interval (CI 95%) were calculated using the Monte Carlo method

Parameters	Answers YES versus NO correct diagnosis				Answers YES versus NO incorrect diagnosis			
	p_{chiQ}	CI 95% lower limit	CI 95% upper limit	YES [%]	p_{chiQ}	CI 95% lower limit	CI 95% upper limit	YES [%]
Smoking	0.002	0.001	0.003	15.6	0.003	0.002	0.005	3.8
Previous stroke	<0.001	0.000	0.000	28.6	<0.001	0.000	0.000	19.2
Hypercholesterolemia	<0.001	0.000	0.000	29.9	0.001	0.000	0.002	11.5
Arrhythmia	0.362	0.353	0.372	44.2	0.003	0.002	0.004	19.2
Arterial hypertension	<0.001	0.000	0.000	74.0	0.556	0.545	0.564	57.7
Diabetes mellitus	0.004	0.003	0.005	32.5	0.003	0.002	0.004	19.2
Preserved orientation	1.000	1.000	1.000	49.4	0.560	0.550	0.565	42.3
Drooping mouth corner	0.903	0.897	0.909	50.6	0.003	0.002	0.004	19.2
Aphasia	0.001	0.001	0.002	68.8	0.169	0.161	0.176	65.4
Hemiplegia	<0.001	0.000	0.000	85.7	1.000	1.000	1.000	50.0
Visual disturbances	0.003	0.002	0.004	16.9	<0.001	0.000	0.000	19.2
Anisocoria	<0.001	0.000	0.000	6.5	<0.001	0.000	0.000	3.8
Conscious	<0.001	0.000	0.000	85.7	<0.001	0.000	0.000	92.3
